# Pre-treatment with *Lactobacillus plantarum* prevents severe pathogenesis in mice infected with *Leptospira interrogans* and may be associated with recruitment of myeloid cells

**DOI:** 10.1371/journal.pntd.0005870

**Published:** 2017-08-25

**Authors:** Hari-Hara Potula, Luciana Richer, Catherine Werts, Maria Gomes-Solecki

**Affiliations:** 1 Department of Microbiology, Immunology and Biochemistry, University of Tennessee Health Science Center, Memphis, Tennessee, United States of America; 2 Immuno Technologies Inc., Memphis, Tennessee, United States of America; 3 Institut Pasteur, Unit Biology and Genetics of the Bacterial Cell Wall, Paris, France; 4 INSERM, Équipe Avenir, Paris, France; Medical College of Wisconsin, UNITED STATES

## Abstract

Recent estimates on global morbidity and mortality caused by Leptospirosis point to one million cases and almost 60,000 deaths a year worldwide, especially in resource poor countries. We analyzed how a commensal probiotic immunomodulator, *Lactobacillus plantarum*, affects *Leptospira interrogans* pathogenesis in a murine model of sub-lethal leptospirosis. We found that repeated oral pre-treatment of mice with live *L*. *plantarum* restored body weight to normal levels in mice infected with *L*. *interrogans*. Pre-treatment did not prevent *L*. *interrogans* access to the kidney but it affected the inflammatory response and it reduced histopathological signs of disease. Analysis of the immune cell profiles in lymphoid tissues of mice pre-treated with *L*. *plantarum* showed increased numbers of B cells as well as naïve and memory CD4+ helper T cell populations in uninfected mice that shifted towards increased numbers of effector CD4^+^ helper T in infected mice. CD8^+^ cytotoxic T cell profiles in pre-treated uninfected and infected mice mirrored the switch observed for CD4+ except that CD8+ memory T cells were not affected. In addition, pre-treatment led to increased populations of monocytes in lymphoid tissues of uninfected mice and to increased populations of macrophages in the same tissues of infected mice. Immunohistochemistry of kidney sections of pre-treated infected mice showed an enrichment of neutrophils and macrophages and a reduction of total leucocytes and T cells. Our results suggest that complex myeloid and T cell responses orchestrate the deployment of monocytes and other cells from lymphoid tissue and the recruitment of neutrophils and macrophages to the kidney, and that, the presence of these cells in the target organ may be associated with reductions in pathogenesis observed in infected mice treated with *L*. *plantarum*.

## Author summary

Leptospirosis is an emerging neglected zoonotic disease with worldwide distribution that affects nearly all vertebrates and causes infection in ~1 million people on a yearly basis. Effective cross-protective vaccines are not available and antibiotic treatment is only effective if used early in the course of infection. In this study we describe how repeated oral treatment of mice with a commonly used probiotic, *Lactobacillus plantarum*, did not completely prevent colonization of the kidney by *Leptospira interrogans* but it did reduce signs and symptoms of leptospirosis. We also analyzed a number of immune cell types in spleen, lymph nodes and kidney after treatment and found that complex responses orchestrate the deployment of phagocytes to the kidney in infected mice. Our results suggest that pre-treatment with *L*. *plantarum* modulates systemic immune responses in a beneficial way in a mammalian host later exposed to *L*. *interrogans* infection.

## Introduction

A recent review on global morbidity and mortality caused by Leptospirosis estimates about 1.03 million cases and 58,900 deaths a year worldwide [1], mostly in resource-poor countries [2][3]. Human leptospirosis is an acute febrile illness with a broad clinical spectrum ranging from mild influenza-like symptoms to severe disease forms characterized by bleeding, jaundice, renal failure, pulmonary hemorrhage and death [2, 3]. Although most leptospirosis patients recover without treatment [3, 4], diagnosis of the disease is hindered by the complexity and insensitivity of serology by the microagglutination test (MAT) in acute infection [5]. Early initiation of antibiotic therapy may thwart disease progression [3]. Hence, practical strategies should prioritize early treatment and prevention to improve outcomes from this spirochaetal zoonosis [6]. Vaccines to prevent human disease exist in some countries and are based in killed whole cell *Leptospira* [3]. However, these vaccines provide only short-time protection, are serovar specific and mostly target leptospiral LPS [2].

*Lactobacillus plantarum* is a Gram-positive bacterium that is known to have immunomodulatory properties [7] and is used as a probiotic usually following high dose repetitive administration regimens [8]. With the long-term goal of using commensal probiotics as vehicles to express *Leptospira* immunogens, we analyzed how repeated pre-exposure treatment of mice with live *L*. *plantarum* affected dissemination of *Leptospira interrogans* to target tissues as well as the ensuing pathology. In the process, we evaluated the immunological mechanisms involved in *Leptospira* pathogenesis.

## Materials and methods

### Animals and ethics statement

Female, 5 week old, C3H/HeJ mice were obtained from The Jackson Laboratory. This study was carried out in accordance with the Guide for the Care and Use of Laboratory Animals of the NIH. The protocol was approved by the University of Tennessee Health Science Center Institutional Animal Care and Use Committee, Animal Care Protocol Application (Permit Number: 14–018).

### Bacterial strains

We used *Lactobacillus plantarum* strain 256 (kindly gifted by Dr. Jos Seegers, Caelus Pharmaceuticals BV), a bacterium Generally Recognized As Safe, to perform oral treatments as described [9], [10] prior to *Leptospira* infection. The strain used in this study (256) was selected from a wide panel of rifampicin-resistant wild-type lactobacilli that were amenable to transformation and persisted in the gut for up to 12 days [8]. Infections of mice were done using 2.5x10^7^ *Leptospira interrogans* serovar Copenhageni strain Fiocruz L1-130 (hamster passage 2, passaged in culture once) in 100-200ul of PBS. Infection dose, culture conditions and spirochete enumeration were described previously [11].

### Oral treatment regimen and infection

**Fig 1:**
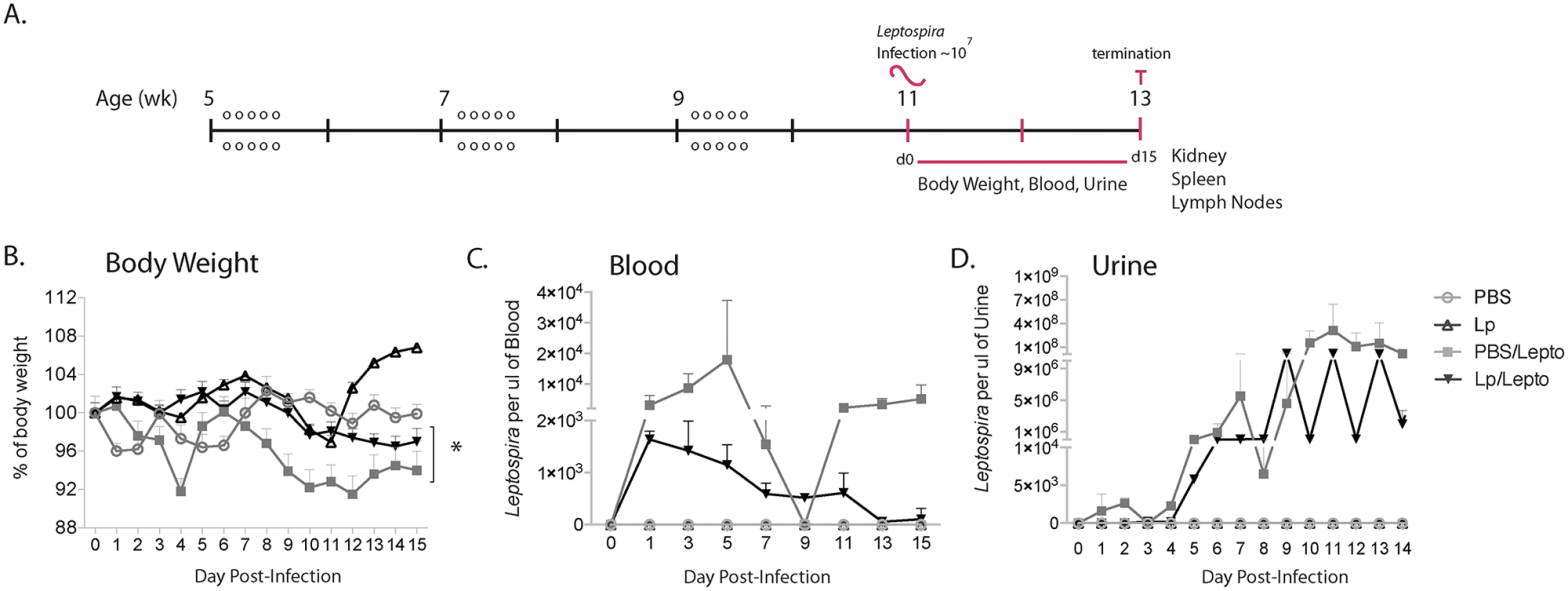
Body weight and *Leptospira* load in body fluids after infection of C3H-HeJ mice pre-treated with *L*. *plantarum*. A. Treatment/infection schedule: groups of 5 week old mice received repeated oral treatments of *L*. *plantarum* (o, n = 30) or PBS and were infected intraperitoneally with *L*. *interrogans* serovar Copenhageni on week 11; uninfected groups of mice were kept as controls. B. Body weight measurements (g) were recorded for 2 weeks post-infection and normalized to 100% on the day of infection (d0). C. Blood and D. urine were collected for determination of the number of *Leptospira* 16s rRNA per ul of sample by qPCR. Statistics: body weight differences between all groups, Two-way ANOVA, p<0.0001; and paired t-test between infected groups * p<0.0001. Number of mice: Lp and Lp/Lepto n = 4 per group, PBS and PBS/Lepto n = 5 per group. Data represents one of two experiments.

Groups of 5-week old mice received 10^10^ CFU of live *L*. *plantarum* strain 256 (Lp) in 100μl of PBS or PBS alone via oral gavage. Mice received treatments daily for five days and two additional boosters every other week for a total of 30 oral treatments over a period of 5 weeks. One week after the final treatment mice were infected intraperitoneally with a sublethal dose of *L*. *interrogans*. Control groups of treated mice were kept uninfected. Mice were monitored for two weeks: daily records were kept for changes in body weight, urine was collected every day and 50μl of blood was collected every other day. At termination, 8-weeks after the start of oral treatments and 2-weeks post infection, blood, kidney, spleen and lymph nodes were collected. Kidney tissue was used for quantification of spirochetes, quantification of immune marker transcripts, histopathology and culture at 30°C for 7 days for determination of spirochetal viability. Peripheral lymph nodes (mandibular, axillary, brachial, inguinal) and spleen were used for immunophenotyping (Fig 1A).

### ELISA

Quantification of total mouse immunoglobulin concentration IgM, IgG, IgG1, IgG2a, IgG3 in mouse serum was done using Ready-Set-Go ELISA kits (eBioscience).

### Quantification of chemokines and cytokines in serum

A panel of chemokines and cytokines, CxCL1 (KC), CxCL2 (MIP-2), CCL5 (RANTES), TNF-α, and IFN-γ were measured in mouse serum (1:2 dilution in assay buffer) with ProcartaPlex Multiplex Immunoassays (eBioscience) according to the manufacturer’s instructions. Samples were processed with xMAP Technology by MagPix Luminex using 50-bead count and analyzed with eBioscience Procarta Plex Analyst software.

### q-PCR and RT-PCR

DNA was extracted from blood, urine, and kidney using a NucleoSpin tissue kit. *Leptospira* was quantified using TAMRA probe and primers (Eurofins) to *Leptospira* 16s rRNA by qPCR. Total RNA was extracted from tissues using an RNeasy mini kit and reverse transcribed using a high-capacity cDNA reverse transcription kit. cDNA was subjected to real-time PCR using primer and TAMRA probes previously described [11]. PCR data are reported as the relative increase in mRNA transcript levels of CxCL1 (KC), CxCL2 (MIP-2), CCL5 (RANTES), TNF-α, IFN-γ, iNOS and ColA1 corrected for by the respective levels of β-actin or to GAPDH. A primer list is provided in supplemental material (S1 Table).

### Flow cytometry

Single cell suspensions of lymphoid tissues were prepared as described [11]. Live/dead cell stain was used to eliminate dead cells. Cells were incubated in Fc blocking for 15 min at 4°C in staining buffer and incubated with the appropriate marker for surface staining in the dark for 30 min at 4°C. Surface markers for T cells (CD3, CD4, CD8, CD62L, CD44) were described previously [11]. The surface marker for B cells was CD-19 conjugated with PercP Cy5.5. For myeloid cells the following lineage surface markers (Tonbo) were used (1:200): CD3 conjugated with pacific blue, MHC-II conjugated with allophycocyanin, CD11b conjugated with phycoerythrin Cy7, CD11c conjugated with allophycocyanin Cy7, and Ly6C conjugated with FITC. Cells were acquired on a BD-LSR II flow cytometer and analyzed using Flow Jo software.

### Histopathology and immunostaining

Kidneys were fixed with 4% paraformaldehyde and processed for histology (paraffin blocks) or immunostaining (5-μm cryosections). For histopathology, tissues were stained by H&E and evaluated for interstitial inflammation and glomerular size and tubular damage under an Axio Zeiss Imager A1 light microscope. Glomeruli were scored by measuring size in 5 fields per sample and averaging groups. Nephritis scores were graded blindly on a scale of 0–5 in a longitudinal section of the organ following previously published criteria [12]. Silver stain of kidney sections were used to visualize *Leptospira* under a light microscope. Fibrosis was evaluated after Masson’s trichrome staining of kidney sections [13] in which 3 randomly chosen fields were digitally analyzed (40x, Photoshop) as a percentage of pixels of the total area. Immunohistochemistry was done at the Translational Pathology Shared Resource, Vanderbilt University Medical Center. Briefly, slides were deparaffinized and heat induced antigen retrieval was performed on the Bond Max using Epitope Retrieval 2 solution for 20 minutes. Sections were blocked at room temperature using saline containing 0.1% BSA and 10% goat serum (Jackson ImmunoResearch Laboratories). The following primary antibodies were used: anti-CD45 (Abcam), anti-neutrophil antibody (NIMP-R14, Abcam), anti-F4/80 (NB600-404, Novus Biologicals), and anti-CD3 (Abcam), and biotinylated secondary antibodies were developed with streptavidin HRP and diaminobenzidine (Vector lab). Blinded quantification of cells expressing the specified marker was done by counting positive cells/total cells in 10 randomly chosen fields (400X) from the cortex and medulla of kidney. At least two sections per kidney were counted for each experiment. Images were taken at 200X, or 400X using a Zeiss microscopy with ZEN software.

### Statistics

Data analysis was done using GraphPad Prism software. Multiple comparisons between groups were done by Two-Way ANOVA. Single comparisons within uninfected and infected groups were analyzed with two-tailed paired t-test, with two-tailed unpaired Mann-Whitney U test (interstitial nephritis), with unpaired t-test with Welch’s correction and with multiple t-tests. α = 0.05 for all tests.

## Results

### Pre-treatments with *L*. *plantarum* restores weight-gain and affects *L*. *interrogans* load in blood and urine

Mice repeatedly treated with a live bacterium, *L*. *plantarum* (Lp), as well as PBS treated controls were infected intraperitoneally with a sub-lethal dose of *Leptospira interrogans serovar Copenhageni* on day 0 and were monitored for 2 weeks. Two additional groups of control mice received oral treatments but were not infected. Body weight loss/gain was recorded daily, blood was collected on alternate days, and urine was collected daily for quantification of number of *Leptospira* 16s rRNA by qPCR (Fig 1A). In the infected groups, PBS treated mice lost a maximum of ~9% weight in specific days (4, 10, 11, 12), whereas mice treated with *L*. *plantarum* lost a maximum of ~4% weight on days 13 and 14. Differences in weight between infected mice treated with *L*. *plantarum* were not different from the uninfected controls. Significant differences were seen between all groups (infected versus uninfected, Two-Way ANOVA p<0.0001) and between infected mice previously treated and untreated with *L*. *plantarum* (t-test p<0.0001) (Fig 1B). Quantification of the number of *Leptospira* in blood and urine from infected mice pre-treated with *L*. *plantarum* (Lp/Lepto) trended towards lower numbers of spirochetes compared to infected controls (PBS/Lepto), although these differences were not statistically significant (Fig 1C and 1D).

### Pre-treatment with *L*. *plantarum* does not prevent colonization of the kidney but it mitigates severe kidney pathology and inflammation

**Fig 2:**
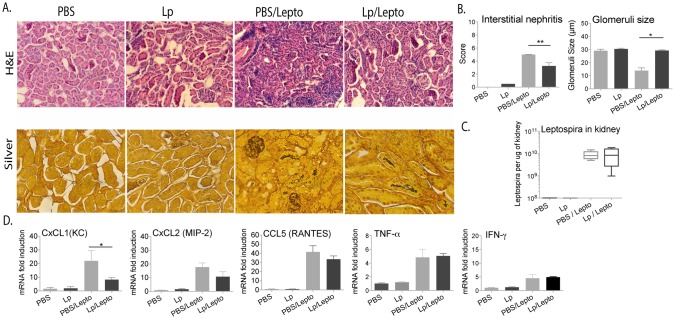
Histopathology, *Leptospira* load and measurement of inflammatory markers in kidney. A. H&E staining (20X) and silver stain (40X) of kidney sections of treated uninfected mice and of mice infected with *Leptospira* after treatment. B. Histopathology was empirically quantified by scoring interstitial nephritis blindly, and glomeruli were scored by measuring size (um) in 5 fields per sample and averaging per group. C. Numbers of *Leptospira* in kidney tissue were quantified by qPCR of 16s rRNA; D. qPCR of pro-inflammatory transcripts (CxCL1, CxCL2, CCL5, TNFα and IFNγ) in kidney. Statistics by unpaired Mann-Whitney U test and by unpaired t test with Welch’s correction between infected groups, B, Interstitial nephritis p = 0.0079, Glomeruli size p<0.0001; D, CxCL1 p = 0.0308, CxCL2 p = 0.0545, CCL5 p = 0.1132, TNFα p = 0.7592, IFNγ p = 0.5788; ** and * p<0.05. Number of mice: Lp and Lp/Lepto n = 4 per group, PBS and PBS/Lepto n = 5 per group. Data represents one of two experiments.

Empirical examination of H&E sections of kidneys collected 2 weeks post infection showed that mice pre-treated with *L*. *plantarum* (Lp/Lepto) had less mononuclear lymphocyte infiltrates and less tubular damage than infected PBS treated controls (PBS/Lepto). Silver stain analysis of the same kidney sections showed morphologically intact *Leptospira* in the tubules of both groups of infected mice pre-treated with PBS or with *L*. *plantarum* (Fig 2A). Quantification of *Leptospira* by qPCR showed about the same numbers of *Leptospira* per ug of kidney tissue from PBS and *L*. *plantarum* treated infected mice (Fig 2C). Culture of the same kidney tissues showed live mobile spirochetes under dark field microscopy and qPCR quantification showed an average of 3-8x10^6^ *Leptospira* in 7-day cultures of kidney from treated mice. We measured the interstitial nephritis scores and the glomeruli size structures in kidney H&E. We found that infected mice pre-treated with *L*. *plantarum* (Lp/Lepto) had lower interstitial nephritis scores than PBS treated infected mice (PBS/Lepto) and that the glomeruli structures of infected mice pre-treated with *L*. *plantarum* (Lp/Lepto) were comparable in size to the uninfected controls. In contrast, infected mice (PBS/Lepto) had decreased glomeruli sizes (Fig 2B). We evaluated *Leptospira*-induced renal inflammation by quantification of mRNA transcription of CxCL1 (KC), CxCL2 (MIP-2), CCL5 (RANTES), TNFα and IFNγ by qRT-PCR. Infected mice pre-treated with *L*. *plantarum* (Lp/Lepto) had lower levels of all three chemokines CxCL1, CxCL2 and CCL5 in kidney than PBS treated infected controls (PBS/Lepto), although only differences in CxCL1 were significant; transcription of cytokines TNFα and IFNγ were not different than controls (Fig 2D).

**Fig 3:**
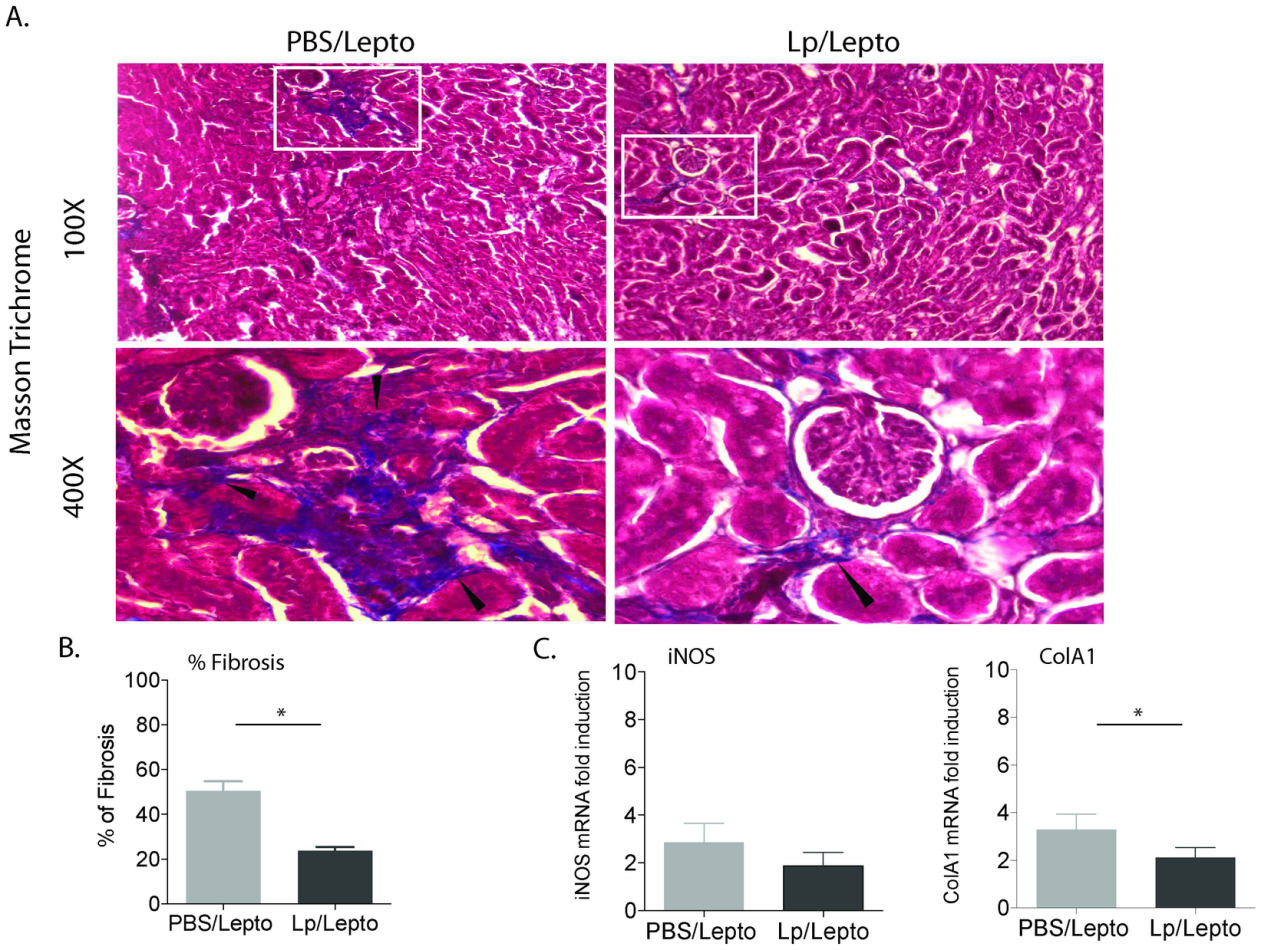
Analysis of renal fibrosis. A. Interstitial collagen deposition (fibrosis, black arrows, 400x) was evaluated by Masson’s trichrome staining of kidney sections from infected mice pre-treated with PBS and with *L*. *plantarum*. B. Digital quantification of fibrosis as determined by the % area (pixels) where blue staining exceeds a threshold. The slides were processed and stained in parallel and images were taken using the same illumination. C. qPCR quantification of fibrosis markers (iNOS and ColA1) in kidney tissue. Statistics between groups by Two-Way ANOVA % fibrosis p = 0.0067 and by unpaired t test with Welch’s correction, iNOS p = 0.1131, ColA1 p = 0.0342; * p<0.05. Number of mice, Lp/Lepto n = 4, PBS/Lepto n = 5. Data represents one of two experiments.

Interstitial collagen deposition (fibrosis) was evaluated using Masson’s trichrome staining of kidney sections from infected mice, which was quantified by digital image analysis (Fig 3). Furthermore, transcription of fibrosis markers (inducible nitric oxide, iNOS and fibroblast activation marker collagen1 A1, ColA1) was quantified by qPCR. Kidney sections from infected mice pre-treated with *L*. *plantarum* had significantly less septa (~23%, Lp/Lepto) of blue-staining fibrillar extracellular matrix expanding tubulointerstitial spaces than infected mice pre-treated with PBS (~50%, PBS/Lepto), (Fig 3A and 3B). Furthermore, infected mice pre-treated with *L*. *plantarum* (Lp/Lepto) had lower levels of fibrosis markers iNOS and ColA1 than infected controls, although only differences for ColA1 were statistically significant (Fig 3C).

### Antibody and chemokine immune responses in serum

**Fig 4:**
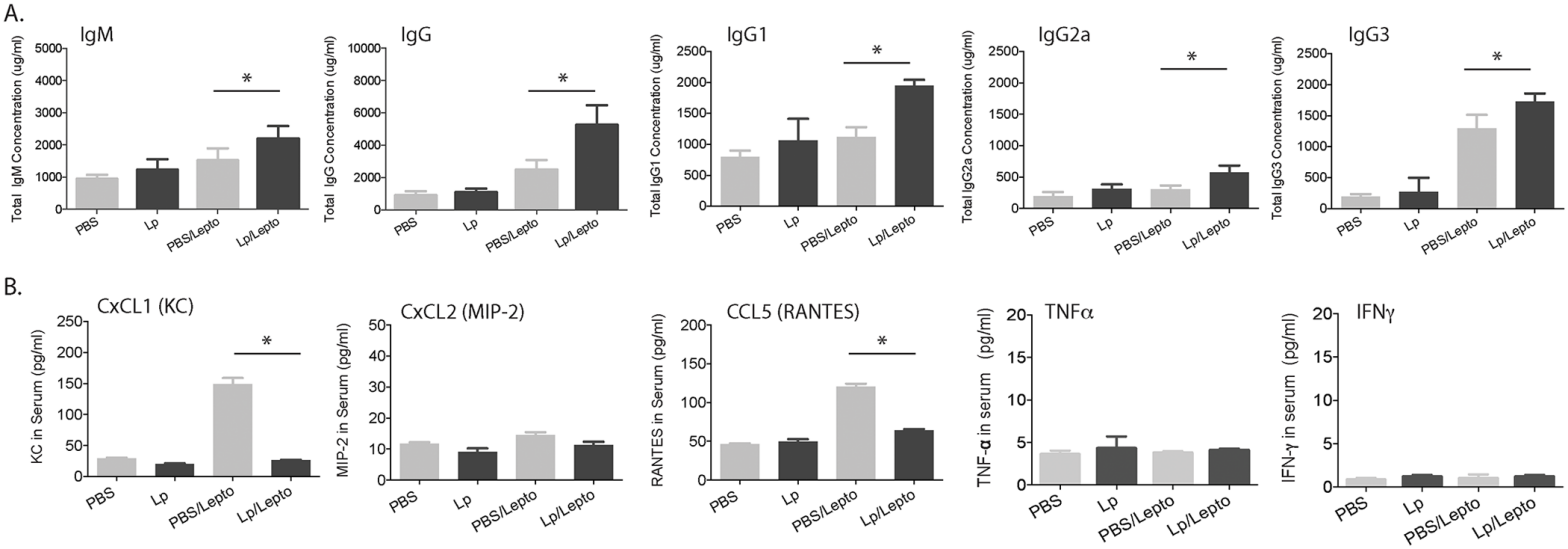
Antibody response and chemokine immunomediators in serum. A. Total concentration of IgM, IgG, IgG1, IgG2a and IgG3 antibodies, and B. CxCL1, CxCL2, CCL5, TNFα and IFNγ, which were quantified by ELISA two weeks post-infection. Statistics: unpaired t test with Welch’s correction between infected groups, Total IgM, p = 0.0457; Total IgG p = 0.0133; Total IgG1 p<0.0001; Total IgG2a p = 0.0083; Total IgG3 p = 0.0085; CxCL1 p = 0.0349; CxCL2 p = 0.0749; CCL5 p = 0.0141; TNFα p = 0.2929; IFNγ p = 0.7142; * p<0.05. Number of mice, Lp and Lp/Lepto n = 4 per group, PBS and PBS/Lepto n = 5 per group. Data shown in A is representative of one of two experiments and data shown in B represents one experiment.

We measured the amount of total antibody in pre-treated uninfected and infected mice (Fig 4). Uninfected mice pre-treated with *L*. *plantarum* (Lp) did not produce higher levels of total antibodies (IgM or IgG, IgG1, IgG2a, IgG3) than controls (PBS). Infected mice pre-treated with *L*. *plantarum* (Lp/Lepto) produced higher amounts of total IgM, IgG, IgG1, IgG2a and IgG3 than the respective infected PBS treated controls (PBS/Lepto) (Fig 4A). Measurements of the concentration of chemokines and cytokines in serum of infected mice pre-treated with *L*. *plantarum* showed significant decreases in chemokines CxCL1 (KC) and CCL5 (RANTES) whereas the concentration of CxCL2 (MIP-2), TNFa and IFNg did not differ from the PBS treated infected controls (Fig 4B).

### Profiles of B and T cells in spleen and peripheral lymph nodes

**Fig 5:**
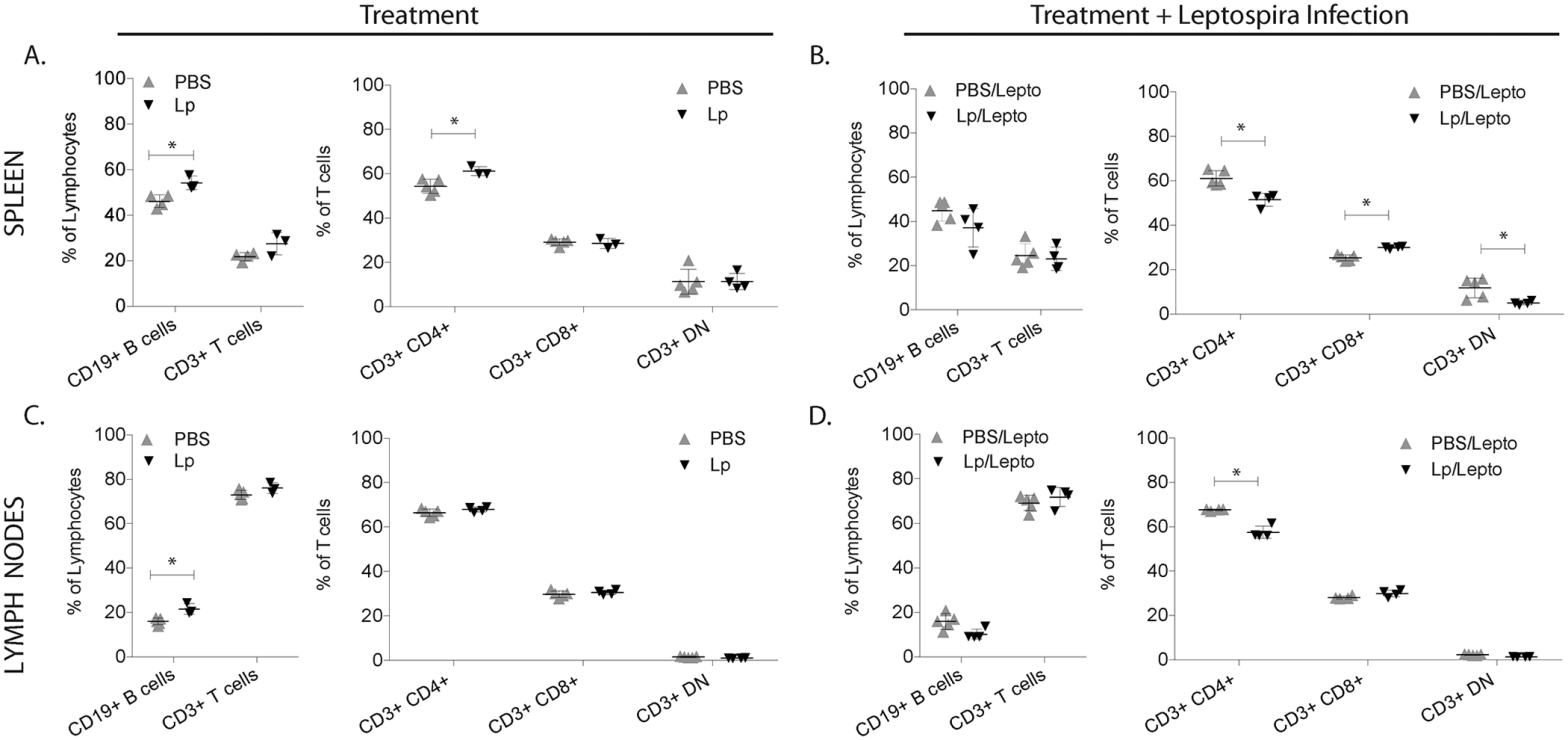
Percentage of B cells, T cells, CD4+ helper T cells, CD8+ cytotoxic T cell and double negative T cell (DN) populations in spleen and lymph nodes. Flow cytometric analysis of immune cells isolated from spleen and peripheral lymph nodes from uninfected and infected mice previously treated with *L*. *plantarum*. Cells were labelled with anti-CD19, anti-CD3, anti-CD4 and anti-CD8 lineage surface markers. Statistics: Multiple t tests: A, Spleen CD19+B cell p = 0.0142, CD4+T cell p = 0.0156; B, Spleen CD4+T cell p = 0.0029, CD8+ T cell p = 0.0002, DN T cell p = 0.0205; C, Lymph Nodes CD19+B cell p = 0.0061; D, Lymph Nodes CD4+ T cell p = 0.0003. A, B, C, D * p<0.05. N = 3 to 5 mice per group x 4 groups (PBS, Lp, PBS/Lepto, Lp/Lepto). Data represents one of two experiments.

**Fig 6:**
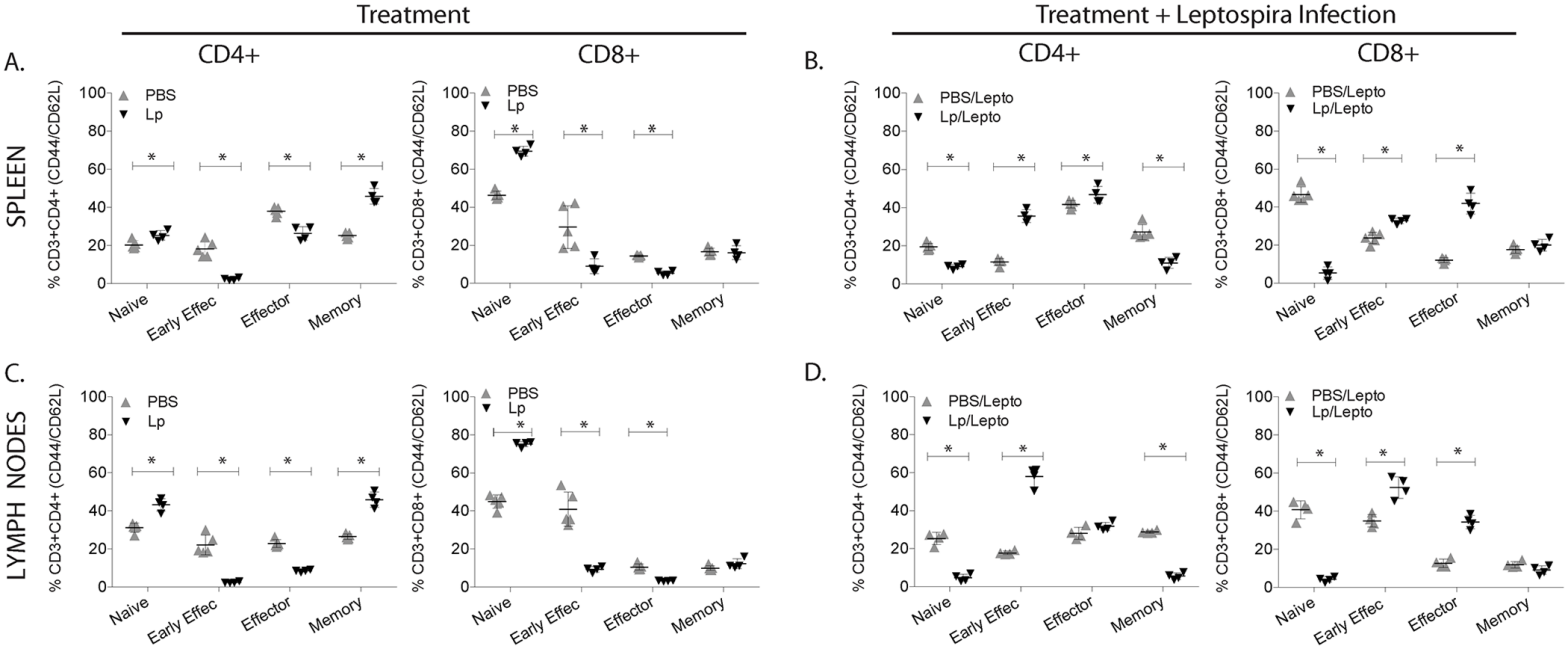
Percentage of naive, effector and memory CD4+ and CD8+ T cell subsets. Flow cytometric analysis of immune cells isolated from spleen and peripheral lymph nodes from uninfected and infected mice previously treated with *L*. *plantarum*. Cells were labelled with anti-CD3, anti-CD4, anti-CD8, anti-CD44 and anti-CD62L lineage surface markers. Statistics by Multiple t tests: A, CD4+ Naive p = 0.0168, Early Effector p = 0.0001, Effector p = 0.0004, Memory p<0.0001; CD8+ Naive p<0.0001, Early Effector p = 0.0106, Effector p<0.0001, Memory p = 0.8073; B, CD4+ Naive p<0.0001, Early Effector p<0.0001, Effector p = 0.0494, Memory p = 0.0002; CD8+ Naive p<0.0001, Early Effector p = 0.0008, Effector p<0.0001, Memory p = 0.2038; C, CD4+ Naive p = 0.0003, Early Effector p = 0.0001, Effector p<0.0001, Memory p<0.0001; CD8+ Naive p<0.0001, Early Effector p = 0.0002, Effector p<0.0001, Memory p = 0.1248; D, CD4+ Naive p<0.0001, Early Effector p<0.0001, Effector p = 0.1018, Memory p<0.0001; CD8+ Naive p<0.0001 Early Effector p = 0.0017, Effector p<0.0001, Memory p = 0.0997; A, B, C, D * p<0.05. N = 3 to 5 mice per group x 4 groups (PBS, Lp, PBS/Lepto, Lp/Lepto). Data represents one of two experiments.

We analyzed B and T cell populations in spleen and peripheral lymph nodes of uninfected and infected mice pre-treated with *L*. *plantarum*, by flow cytometric analysis (Fig 5). Uninfected mice pre-treated with *L*. *plantarum* (Lp) had significant increases of the percentages of B cells in spleen and in lymph nodes, and of CD4+ T cells in spleen (Fig 5A and 5C). Infected mice pre-treated with *L*. *plantarum* (Lp/Lepto) responded with a significant decrease of CD4+ T cells in spleen and in lymph nodes, an increase of CD8+ T cells in spleen and a decrease of Double Negative (DN) T cells in spleen, whereas B cell populations were not affected in both tissues (Fig 5B and 5D). We further analyzed CD4^+^ T helper and CD8+ cytotoxic T cell subsets by labeling naïve (CD62L^+^), early effector (CD62L^−^/CD44^−^), effector (CD44^+^) and memory T cells (CD62L^+^/CD44^+^) in uninfected and infected mice pre-treated with *L*. *plantarum* (Fig 6). Analysis of helper cells revealed that treatment with *L*. *plantarum* (Lp) led to an increase of naïve and memory T cells and to a decrease of early effector/effector in spleen and lymph nodes of uninfected mice (Fig 6A and 6C), whereas infected mice pre-treated with *L*. *plantarum* (Lp/Lepto) shifted CD4+ T cell responses toward an increase in early effector/effector and a decrease of naïve and memory T cells in both tissues (Fig 6B and 6D). Analysis of CD8^+^ cytotoxic T cell profiles in pre-treated uninfected and infected mice mirrored the switch observed for CD4+ except that CD8+ memory T cell populations were not affected.

### Profiles of myeloid cells in spleen and peripheral lymph nodes

**Fig 7:**
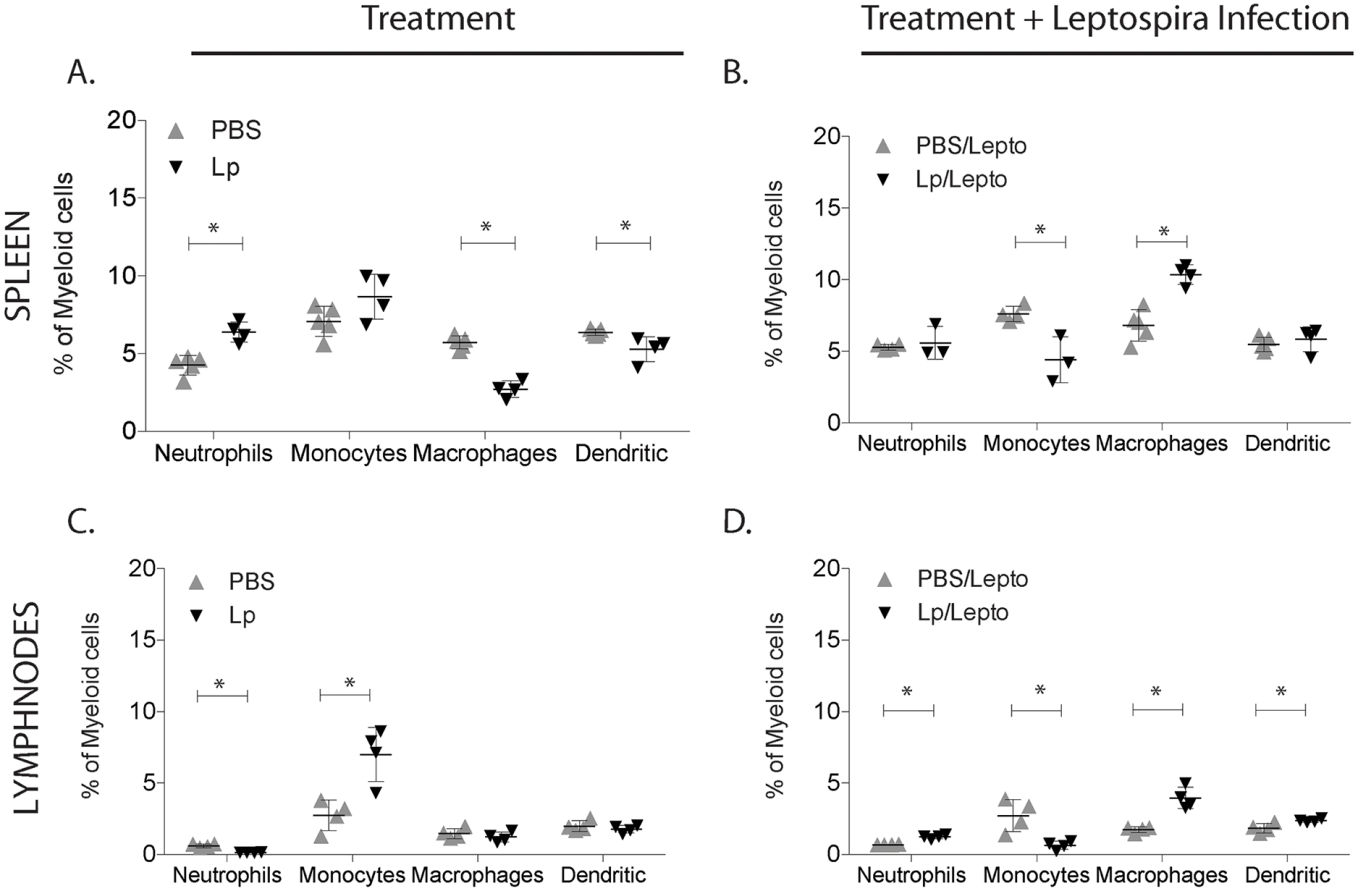
Percentage of neutrophils, monocytes, macrophages and dendritic cell populations in spleen and lymph nodes. Flow cytometric analysis of myeloid cells isolated from spleen and peripheral lymph nodes from uninfected and infected mice previously treated with *L*. *plantarum*. Cells were labeled with anti-MHC II, anti-CD11b, anti-CD11c and anti-Ly6C lineage surface markers. Statistics by Multiple t tests: A, neutrophils p = 0.0015, monocytes p = 0.0900, macrophages p<0.0001, dendritic p = 0.0219; B, neutrophils p = 0.6008, monocytes p = 0.0122, macrophages p = 0.0007, dendritic p = 0.4653; C, neutrophils p = 0.0003, monocytes p = 0.0077, macrophages p = 0.3968, dendritic p = 0.3892; D, neutrophils p = 0.0002, monocytes p = 0.0114, macrophages p = 0.0012, dendritic p = 0.0232; A, B, C, D * p<0.05. N = 3 to 5 mice per group x 4 groups (PBS, Lp, PBS/Lepto, Lp/Lepto). Data represents one of two experiments.

We further evaluated inflammatory immune responses by profiling four subsets of myeloid cell populations in spleen and in peripheral lymph nodes of uninfected and infected mice pre-treated with *L*. *plantarum*, by flow cytometric analysis using MHC II, CD11b, CD11c, Ly6C markers (Fig 7). Uninfected mice treated with *L*. *plantarum* (Lp) had marked increases of neutrophils (MHC II^−^ CD11b^+^) in spleen and monocytes (MHC II^−^ CD11b^+^ Ly6C^+^) in peripheral lymph nodes, and decreased numbers of macrophages (MHC II^+^ CD11b^+^) and dendritic cells (MHC II^+^ CD11c^+^) in spleen (Fig 7A and 7C). Infected mice pre-treated with *L*. *plantarum* (Lp/Lepto) responded with a significant decrease of monocytes and an increase of macrophages in both tissues (Fig 7B and 7D).

### Pre-treatment with *L*. *plantarum* leads to recruitment of neutrophils and macrophages to the kidney in infected mice

**Fig 8:**
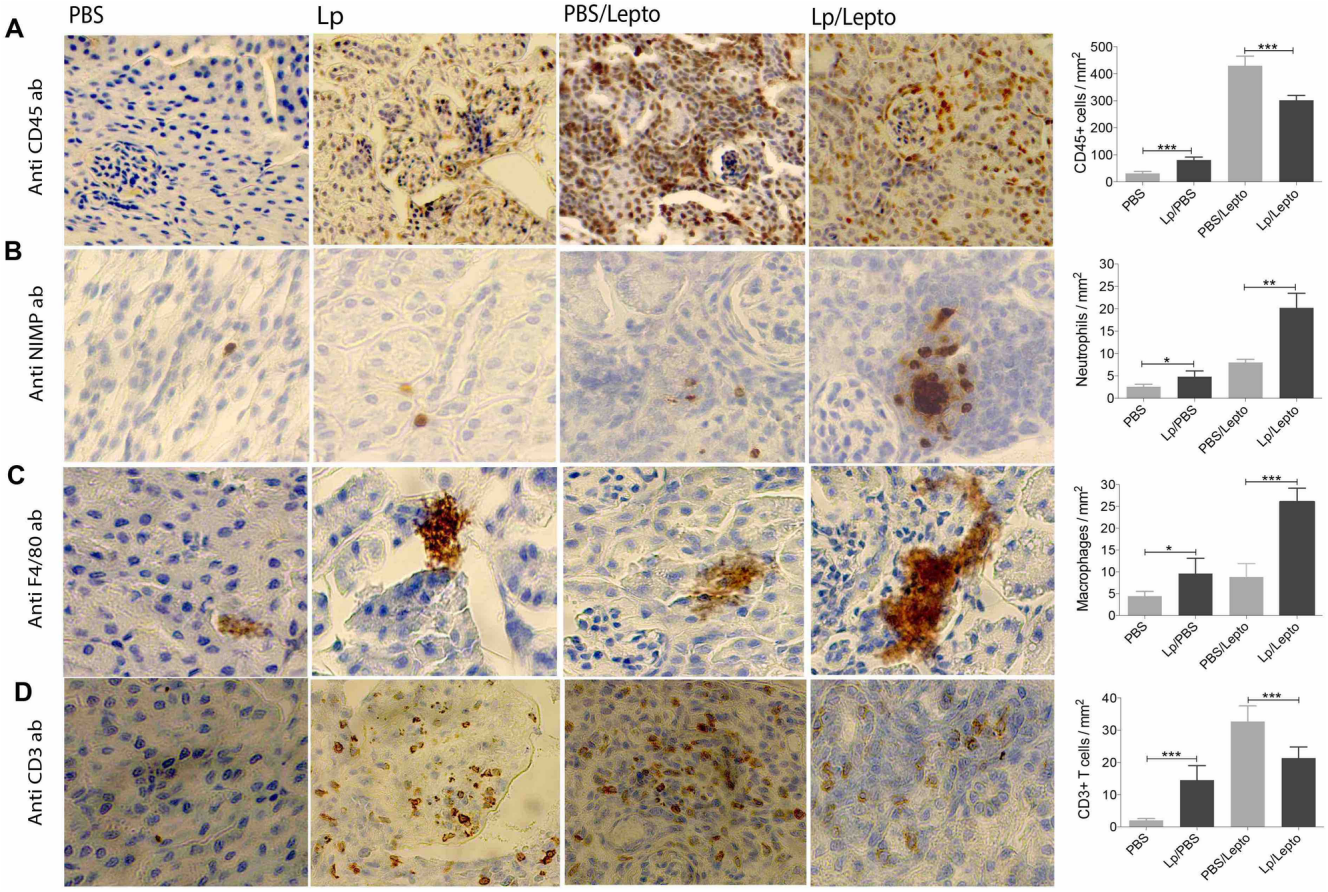
Oral treatment with *L plantarum* leads to recruitment of myeloid cells in kidney. Immunostaining of kidney sections from groups of treated mice, in the presence or absence of *Leptospira* infection using various leukocyte markers. We determined the number of CD45+, NIMP-R14+, F4/80+ and CD3+ T cells per millimeter squared of kidney. Data are mean SEM of 4–5 mice per group. Scale bar represents 200X for (A) and 400x for (B), (C) and (D). Statistics by two-tailed paired t-test *p < 0.05, ** p < 0.01, *** p < 0.001. Data is representative of one of two experiments.

We analyzed whether *L*. *plantarum* treatment promotes recruitment of distinct leucocyte populations to the target organ in the presence and absence of *Leptospira* infection by immunohistochemistry of kidney sections using CD45^+^, NIMP-R14^+^, F4/80^+^ and CD3^+^ to stain total leucocytes, neutrophils, macrophages and T cells, respectively (Fig 8). In the absence of infection, kidneys of mice pre-treated with *L*. *plantarum* (Lp) had moderately increased numbers of myeloid cells compared to PBS treated controls (PBS). However, in the presence of *Leptospira* infection numbers of CD45+ total leucocytes and CD3+ T cells increased by 10 fold (PBS/Lepto); these numbers were reduced by a third in kidneys of infected mice pre-treated with *L*. *plantarum* (Lp/Lepto), (Fig 8A and 8D). A striking observation was that infected mice pre-treated with *L*. *plantarum* (Lp/Lepto), which we had previously determined to have less kidney damage (Figs 2 and 3), had a marked increased in NIMP-R14^+^ neutrophils and F4/80^+^ macrophages (at least 2 fold higher) as compared to infected PBS-treated controls (PBS/Lepto), (Fig 8B and 8C). In the kidney of these mice (PBS/Lepto), which we previously determined to have more damage than *L*. *plantarum* treated infected mice, we detected a large number of infiltrating leucocytes looking like PMN that are not positive for NIMP-R14.

## Discussion

New technologies are being used to advance knowledge of host immunity to pathogenic *Leptospira* species [14–21]. One aspect of the work done in our laboratory is the design of molecular tools and development of oral delivery vehicles for prevention or therapeutic management of infectious diseases [9, 10, 22–24]. There is scientific evidence supporting the health effects generally attributed to consumption of commensal probiotics with a number of studies discussing application to diseases caused by gastrointestinal, respiratory and urogenital bacterial infections [1, 25, 26]. In this study, we evaluated if a commensal probiotic immunomodulator, *Lactobacillus plantarum*, can be used for prevention of leptospirosis.

Analysis of *Leptospira* burden and disease scores using the sublethal C3H/HeJ mouse model of infection [11] showed that repeated oral pre-treatments with live *L*. *plantarum* restored weight gain and affected *L*. *interrogans* burdens in blood and urine, but it did not prevent colonization of the kidney as demonstrated by the detection of *Leptospira* DNA by qPCR, visualization of morphologically intact spirochetes in silver stains and culture of live *L*. *interrogans* from kidney tissues in infected mice pre-treated with *L*. *plantarum*. Kidney H&E histopathology data showed that infected mice pre-treated with *L*. *plantarum* (Lp/Lepto) had less mononuclear lymphocyte infiltrates, less tubular damage and less interstitial nephritis scores than infected controls pre-treated with PBS (PBS/Lepto). Glomeruli size in kidney of infected mice pre-treated with *L*. *plantarum* (Lp/Lepto) were about the same size as Lp and PBS uninfected controls [27][11, 28]. Further analysis of immune marker transcripts in kidney (CxCL1/KC, CXCL2/MIP-2, CCL5/RANTES, TNFα and IFNγ) showed that infected mice pre-treated with *L*. *plantarum* produced less pro-inflammatory chemokines KC, MIP-2 and RANTES known to be upregulated during infection [13, 29–31][11] although transcription of cytokines TNFα and IFNγ was not affected. A decrease in the same chemokine markers was also observed in blood. Differences in KC were significant in kidney and blood, and differences in RANTES were significant only in blood. Furthermore, renal fibrosis was analyzed by staining of interstitial collagen deposition of kidney sections and by molecular quantification of mRNA transcripts of fibroblast activation marker (collagen1 A1, ColA1) and inducible nitric oxide (iNOS) which play a role in *Leptospira*-induced interstitial nephritis [13] and induction of kidney fibrosis [16]. Infected mice pre-treated with *L*. *plantarum* (Lp/Lepto) had ~50% less fibrosis and produced less transcripts of ColA1 than infected mice pre-treated with PBS (PBS/Lepto). These results suggest that repeated oral treatment with live *L*. *plantarum* prevented upregulation of collagen 1A1 mRNA and transcription of pro-inflammatory chemokines which may have reduced accumulation of collagen in the tubulointerstitial spaces, thereby rescuing severe kidney pathology. Moreover, since the bacterial load in kidney is equivalent in *L*. *plantarum* (Lp/Lepto) and PBS treated infected mice (PBS/Lepto), these data suggest that renal lesions may be triggered by the host inflammatory immune response rather than by direct presence of *Leptospira* toxins or metabolism. However, we previously showed that renal fibrotic lesions occur only when live *Leptospira*, not remnant antigens, are present in the kidney and that the fibrosis is not proportional to the bacterial load [16]. We also showed that fibrosis could occur in absence of B or T cells [16]. Thus, the question remains about the factors leading to renal lesions in infected mice, that we show here to be reduced by oral treatments with *L*. *plantarum*.

It is generally understood that protection against *Leptospira* infection is most likely driven by humoral antibody responses. We analyzed the contribution of antibody responses to *Leptospira* infection in mice pre-treated with *L*. *plantarum* and with PBS. We detected significant increases in total IgM, IgG, IgG1, IgG2a and IgG3 only in infected mice pre-treated with *L*. *plantarum*. High amounts of IgG1 and IgG3 suggest that initial neutralization of *Leptospira* may be mediated by IgM with help of IgG1 and IgG3 which may have affected bacterial loads in blood and urine. However, this response was either late or not robust enough to prevent access of *Leptospira* to the kidney, since equivalent loads of *Leptospira* were measured in kidney of *L*. *plantarum* and PBS treated infected mice. It is known that pathogenic *Leptospira* reach the kidney in the very first hours post intraperitoneal infection to escape blood defenses [14, 15].

Analysis of the effect of *L*. *plantarum* treatments in adaptive immune response shows that uninfected mice had increased numbers of B cells, increased numbers of naïve and memory CD4+ T cells and lower numbers of effector CD4+ cells in spleen and lymph nodes. In contrast, the immune cell profiles of infected mice pre-treated with *L*. *plantarum* shifted toward a decreased number naïve and memory CD4+ helper T cells and to an increased number of effector CD4+ T cells in both tissues, whereas B cell populations remained the same as infected PBS treated controls. Changes in populations of CD8+ cytotoxic T cells mimicked CD4+ helper T cells except that CD8+ memory was not affected. These results suggest that infection with *Leptospira* engages a systemic immune response spearheaded by effector T helper and cytotoxic T cells in mice pretreated with *L*. *plantarum* and that memory T cells do not appear to be engaged. These results are consistent with findings in other studies in which depletion or absence of T cells led to severe pathological lesions in kidney in C3H/HeJ mice [32] and worse kidney lesions in C57BL/6J [14].

A striking result observed was that, in the absence of infection, treatments with *L*. *plantarum* led to an increase of neutrophils and a decrease of macrophages and dendritic cells in spleen, and to an increase of monocytes in peripheral lymph nodes, whereas *Leptospira* infection of mice pre-treated with *L*. *plantarum* led to decreased populations of monocytes and increased populations of macrophages in both tissues. Immunohistochemistry of sections of kidney of infected mice pre-treated with *L*. *plantarum* showed an overall decrease of CD45+ total leucocytes and CD3+ T cells which is consistent with the lower numbers of mononuclear lymphocyte infiltrates observed using H&E staining. Furthermore, we observed an enrichment of neutrophils and macrophages in this target tissue. We speculate that the enrichment of populations of monocytes in peripheral lymphoid tissue after treatment with *L*. *plantarum* may result in deployment of larger numbers of these cells to target organs after *Leptospira* infection, which could explain the higher numbers of macrophages we measured in spleen, lymph nodes and in the kidney. In kidney of infected mice treated with PBS we detected a number of leucocytes that failed to stain for the NIMP-R14 marker suggesting that *Leptospira* infection does not trigger recruitment of PMNs, which were abundantly found in kidneys of mice pretreated with *L*. *plantarum*. If these PMN were phagocytic, we would have expected a decrease in the number of *Leptospira* in the kidney of *L*. *plantarum* treated group, which was not the case. Neutrophil enrichment in lymphoid tissue after *L*. *plantarum* treatment could potentially explain homing of these cells to the kidney after *Leptospira* infection that could result in entrapment of *Leptospira* via NETosis. Neutrophil extracellular traps have been shown to be involved in the innate immune response to infection with *Leptospira interrogans* Fiocruz L1-130 [19] also used in our study. However, in Scharrig et al. depletion of PMN resulted in a decrease of the number of *Leptospira* in blood 3 days post infection and in kidney 15 days post infection, even though nephritis remained the same in non depleted mice, suggesting that PMN do not play a role in nephritis [19]. In our study, the presence of both macrophages and neutrophils in kidney appears to be associated with the decrease of pathogenic activity of *Leptospira* in this target tissue. This is paradoxical considering the common notion that both macrophages and neutrophils are supposed to be the cells producing nitric oxide and fibrogenic components, which have negative effects on host cells [13]. A reduction of T cells in kidney could imply that T cell signaling is important to orchestrate systemic immune responses to infection with *Leptospira* but these cells may not be directly involved in resolving infection in the kidney.

Our results suggest that in mice infected with *L*. *interrogans*, pre-treatment with *L*. *plantarum* triggers a complex myeloid and T cell response that orchestrates the deployment of monocytes from lymphoid tissue and the recruitment of neutrophils and macrophages to kidney. Further, the presence of these myeloid cells in kidney may be associated with the reductions in pathogenesis observed. Future studies to analyze the effect of *L*. *plantarum* in post-exposure treatments could also shed light on the possible use of commonly available probiotics as alternative palliative care for leptospirosis.

## Supporting information

S1 Table Colla1, fibroblast activation marker collagen1 A1; GAPDH, glyceraldehyde-3-phosphate dehydrogenase; iNOS, inducible nitric oxide; CxCL1/KC, chemokine (C-X-C motif) ligand 1 or Keratinocyte Chemoattractant (KC); CxCL2/MIP-2, chemokine (C-X-C motif) ligand 2 or macrophage inflammatory protein 2; CCL5/RANTES, chemokine (C-C motif) ligand 5 or regulated on activation, normal T cell expressed and secreted; IFN-G, interferon gamma; TNF-A, tumor necrosis factor alpha. (DOC)
