# Supplementary material for: Pre-treatment with Lactobacillus plantarum prevents severe pathogenesis in mice infected with Leptospira interrogans and may be associated with recruitment of myeloid cells
Source: PLoS Negl Trop Dis. 2017 Aug 25;11(8):e0005870. doi: 10.1371/journal.pntd.0005870 (PMC5589268; doi:10.1371/journal.pntd.0005870)
Supplement: S1 Table — (DOC) [file pntd.0005870.s001.doc]

Supplementary Table 1

S1 Table. Primers and probes used for real time PCR.

| Col1a1-1F -Forward | 5’ TAAGGGTACCGCTGGAGAAC 3’ |
| --- | --- |
| Col1a1-1R-Reverse | 5’ GTTCACCTCTCTCACCAGCA 3’ |
| FAM TAMRA- Col1a1-1P | 5’ AGAGCGAGGCCTTCCCGGAC 3’ |
| GAPDH-Forward | 5’ GTCGGTGTGAACGGATTTGGCCG 3’ |
| GAPDH -Reverse | 5’ TCAATGAAGGGGTCGTTGAT 3’ |
| FAM TAMRA- GAPDH | 5’ GCCTGGTCACCAGGGCTGCCATTTG 3’ |
| iNOS-Forward | 5’ GCTGGGCTGTACAAACCTTC 3’ |
| iNOS-Reverse | 5’ GCATTGGAAGTGAAGCGTTTC 3’ |
| FAM TAMRA- iNOS | 5’ GGCAGCCTGTGAGACCTTTGAT 3’ |
| KC 1F-Forward | 5’ CGAGGCTTGCCTTGACCCTGAA 3’ |
| KC 1R-Reverse | 5’ GGGACACCTTTTAGCATCTT 3’ |
| KC-TAMRA | 5’ CCCTTGGTTCAGAAAATTGTCCA 3’ |
| MIP-2 Forward | 5’ TGACTTCAAGAACATCCAGAGCTT 3’ |
| MIP-2 Reverse | 5’ CTTGAGAGTGGCTATGACTTCTGTC 3’ |
| MIP-2 TAMRA | 5’ TGACGCCCCCAGGACCCCA 3’ |
| RANTES Forward | 5’ AGTGCTCCAATCTTGCAGTCGT 3’ |
| RANTES Reverse | 5’ CTTCTTCTCTGGGTTGGCACACACTT 3’ |
| RANTES TAMRA | 5’ TTGTCACTCGAAGGAACCG 3’ |
| IFN-G Forward | 5’ CAAGTGGCATAGATGTGGAAGAAA 3’ |
| IFN-G Reverse | 5’ CTGGCTCTGCAGGATTTTCA 3’ |
| IFN-G TAMRA | 5’ GGAGGAACTGGCAAAAGGATGGTGAC 3’ |
| TNF-A Forward | 5’ CACACTCAGATCATCTTCTCAAAAT 3’ |
| TNF-A Reverse | 5’ AAGGTACAACCCATCGGCTGGCA 3’ |
| TNF-A TAMRA | 5’ AGCCTGTAGCCCACGTCGTAGCAAAC 3’ |
| β-actin Forward | 5’ CCACAGCTGAGAGGGAAATC 3’ |
| β-actin Reverse | 5’ CCAATAGTGATGACCTGGCCG 3’ |
| β-actin TAMRA | 5’ GGAGATGGCCACTGCCGCATC 3’ |
